# 25-hydroxyvitamin D is a predictor of COVID-19 severity of hospitalized patients

**DOI:** 10.1371/journal.pone.0268038

**Published:** 2022-05-03

**Authors:** Nguyen N. Nguyen, Muppala N. P. Raju, Briget da Graca, Dapeng Wang, Nada A. Mohamed, Manohar B. Mutnal, Arundhati Rao, Monica Bennett, Matthew Gokingco, Huy Pham, Amin A. Mohammad

**Affiliations:** 1 Department of Pathology, Baylor Scott & White Central Texas, Temple, Texas, United States of America; 2 Baylor Scott & White Research Institute, Dallas, Texas, United States of America; Holbaek Sygehus, DENMARK

## Abstract

**Objectives:**

Studies investigating the association between vitamin D and severity of COVID-19 have mixed results perhaps due to immunoassay assessment of total 25-hydroxyvitamin D (tD) (the sum of 25-hydroxyvitamin-D2 [25-OH-D2] and 25-hydroxyvitamin-D3 [25-OH-D3]). Liquid chromatography tandem mass spectrometry (LC-MS/MS) has high analytical specificity and sensitivity for 25-OH-D2 and 25-OH-D3, and thus enables a more accurate assessment of impact on COVID-19 outcomes.

**Methods:**

We established reference intervals for 25-OH-D3 and tD using LC-MS/MS. 25-OH-D2, 25-OH-D3 and tD were quantitated for 88 COVID-19 positive and 122 COVID-19 negative specimens. Chi-square or Fisher’s exact tests were used to test associations in binary variables. T-Tests or Wilcoxon rank sum tests were used for continuous variables. Cox proportional hazards were used to test associations between 25-OH-D3 or tD levels and length of stay (LOS). For mortality and ventilation, logistic regression models were used.

**Results:**

COVID-19 patients with deficient (<20 ng/mL) levels of 25-OH-D3 had significantly longer LOS by 15.3 days. COVID-19 P patients with deficient (<20 ng/mL) and insufficient (<30 ng/mL) of tD had significantly longer LOS by 12.1 and 8.2 days, respectively. Patients with insufficient levels of tD had significantly longer LOS by 13.7 days. COVID-19 patients with deficient serum 25-OH-D3 levels had significantly increased risk-adjusted odds of in-hospital mortality (OR [95% CI]: 5.29 [1.53–18.24]); those with insufficient 25-OH-D3 had significantly increased risk for requiring ventilation during hospitalization was found at LCMS insufficient cutoff (OR [95% CI]: 2.75 [1.10–6.90]).

**Conclusions:**

There is an inverse relationship of 25-hydroxyvitamin D levels and hospital LOS for COVID-19 patients. Vitamin D status is a predictor for severity of outcomes. LCMS results are useful for assessing the odds of mortality and the need for ventilation during hospitalization.

## Introduction

Humans depend on fat-soluble vitamin D for many physiologic functions and vitamin D has been found to influence over 200 different genes [1, 2]. Despite widespread fortification in foods, prevalence serum 25-hydroxyvitamin D deficiency less than 20 ng/mL was found in one-third of American populations [3]. Vitamin D status is assessed via the biomarker 25-hydroxyvitamin D because it is a highly available metabolite in the serum with a half-life greater than 2 weeks [4]. The normal healthy adult serum reference interval for total 25-hydroxyvitamin D (tD), measured as the sum of 25-hydroxyvitamin-D2 (25-OH-D2) and 25-hydroxyvitamin-D3 (25-OH-D3), is 30 to 60 ng/mL as previously determined by immunoassay validation. Studies show strong correlation between vitamin D deficiency and progression of cancers, cardiovascular and autoimmune diseases, poorer outcomes following surgery, decreased sensitivity to insulin, increased influenza infection, and worsening of COVID-19 disease and symptoms [1, 5, 6].

Recently, a pilot clinical trial demonstrated that administration of a high dose of Vitamin D3 significantly reduced the need for ICU treatment of COVID-19 patients [7], and a second report with limited case numbers claimed that high dose Vitamin D therapy can shorten length of stay (LOS) for COVID-19 patients [8]. However, other studies have not found any correlation between tD and COVID-19 outcomes following adjustment for confounding variables [9]. None of these published studies evaluated levels of the two components that constitute tD separately, probably due to lack of liquid chromatography tandem mass spectrometry (LCMS) instrumentation, which is typically not found in the clinical laboratories. The results they report are based on immunoassay methodologies, which despite having high analytical sensitivity and precision, are not always accurate.

As a regional tertiary academic medical center in central Texas, we perform around 1000 COVID-19 tests daily. We have a census of around 50 patients admitted at any given time. This, in addition to the availability of Cascadion Analyzer [10, 11], provided an opportunity to study 25-OH-D2 and 25-OH-D3 levels in COVID-19 positive patients and investigate their relationships to disease severity, as indicated by LOS, need for ventilation, and mortality, which may help resolve discordant results reported in previous studies.

## Materials and methods

### Specimens

This retrospective study was approved by the Baylor Scott and White, Research Institute’s Institutional Review Board (IRB, ref: 354589), with a waiver of informed consent. A total of 210 anonymized and to be discarded excess serum samples collected between 7/15/2020 to 10/15/2020 from patients age ≥18 years were used. Each patient had a real time reverse transcription polymerase chain reaction result (rtPCR, Cepheid, Sunnyvale, CA) for SARS-COV2 prior to testing 25-hydroxyvitamin D levels. Of the 210 samples, 88 were from admitted in-patients with confirmed positive for COVID-19 by rtPCR, while the remaining 122 were from outpatients confirmed negative for COVID-19 by rtPCR.

Test validation was done on 392 to be discarded, anonymized and frozen at -20°C serum samples. These samples were collected from out-patients for evaluation of Vitamin D status. A one milliliter aliquot of residual specimen was stored at –20°C until testing was performed. Before testing, the samples were thawed, vortexed, re-centrifuged and pipetted into Cascadion sample cups. All samples were collected in vacutainer tubes with clot activator (Becton Dickinson and Company, Franklin Lakes, NJ). After 30 minutes of clotting at ambient condition, the samples were spun for 10 min according to manufacturer’s protocol.

### Instrumentation

The Cascadion™ SM Clinical Analyzer (ThermoFisher Scientific, Waltham, MA) is an FDA listed class II exempt system. It is an all-in-one random access system consisting of robotic sampling, centrifugation, liquid chromatography, and tandem mass spectrometry. The FDA approved assay kit is able to quantitate levels of 25-OH-D2 and 25-OH-D3 separately [7]. The kit includes a six calibrator set, three controls, and deuterated internal standards. A sample volume of 50 μl is dispensed into the extraction vessel along with 200 μl of internal deuterated analogs 25-OH-D2-d3 and 25D3-d6 in acetonitrile and ethanol solution. The samples are mixed and centrifuged to separate precipitates. Supernatant volume of 65 μl is injected into the Quick Connect Cartridge which uses patented TurboFlow technology to remove large residual molecules and separate analytes respectively. The analytes are ionized by heated electrospray ionization at the mass spectrometer source. Positive ion mode and selected reaction monitoring of mass to charge (m/z) ions via triple quadrupole analyzer, achieving a unit resolution of 0.7 Da full width at half maximum. Precursor ions are selected in the first quadrupole, fragmented by argon gas in the second quadrupole, and selected in the third quadrupole. An electron multiplier amplifies selected signals, which are converted digitally by the computer. Peak responses are calculated into concentration levels via interpolation from peak area ratios of calibrator to deuterated analogs. Ion ratios are monitored for unique fragmentation characteristics.

### Assay validation

Calibration linear regression coefficient criteria r > 0.99 were verified monthly. Linearity was evaluated using Clinical & Laboratory Standards Institute (CLSI) standard EP6-A using spiked samples. Spike recovery was evaluated according to CLSI standard C62-A; pooled donor samples were used as matrix in these experiments. Precision experiments were done on two replicates of each control twice daily over three months and evaluated according to CLSI standard EP05-A3 on MS-Excel (Microsoft, Redmond, WA). Daily quality control was done by batching three controls of 25-OH-D2 and 25-OH-D3 at three levels, with each control acceptable within ± 10% from the expected levels. We performed proficiency testing using the Total Vitamin D and the Accuracy-based Vitamin D kits from College of American Pathologists (CAP, Washington, DC). Interference was evaluated using CLSI standard EP07 for endogenous or exogenous substances. The samples were spiked with high concentration of potentially interfering compounds at medical decision points and percent bias was calculated [12].

Correlation experiments were done by using i) 120 samples from the Vitamin D Standardization-Certification Program (VDSCP) (CDC, Atlanta GA) and comparing the Cascadion results with those reported by VDSCP using liquid chromatography tandem mass spectrometry and ii) 392 frozen serum samples from patients who were evaluated for Vitamin D deficiency using both the Cascadion LC-MS/MS assay and the tD assay on Architect i2000 immunoassay analyzer (Abbott Laboratories, Chicago, IL). The immunoassay reference intervals for tD were previously established from samples collected via direct and a priori sampling from out-patient clinic patients who had no active Vitamin D supplements listed in medical records. These patients were collected prior to the COVID-19 pandemic. None of these patients had abnormal parathyroid hormone, calcium or thyroid stimulating hormone levels, or serious co-morbidities such as renal disease, heart disease, cancer, or non-COVID-19 infectious diseases. We used the universal consensus for deficient cutoff at 20 ng/mL and insufficient cutoff at 30 ng/mL [13–15]. Area under the receiver operating curve (AUC) as well as sensitivity and specificity were calculated for tD and 25-OH-D3 and their associated cutoffs for the mortality outcome.

### Record & statistics

Three independent assessors reviewed patient charts on electronic medical records for relevant age, gender, clinical conditions, total LOS in the hospital, need for ventilation, and in-hospital mortality. Data were tabulated in an MS-Excel spreadsheet. Continuous data were summarized using means and 95% confidence intervals. Categorical variables were summarized using counts and percentages. Chi-square test (or Fisher’s exact test for low counts) were used to test for associations in binary variable. Wilcoxon rank-sum test was used to test for differences in continuous variables between two groups. Statistical significance was determined when p-value ≤0.05. Comparison of LOS by vitamin D level was performed using Cox proportional hazards (CPH) which censored patients who died prior to discharge. Hazard ratios are interpreted as increased hazard of an additional day in the hospital for patients with 25-OH-D3 and tD levels above or below cutoffs. Multiple logistic regression was performed to assess a difference in mortality rates between groups. The odds ratio is interpreted as the increased odds of death for patients with deficient levels of 25-OH-D3 and tD. Both CPH and logistic models were adjusted for age, sex, and body mass index (BMI). Neither interactions nor non-linear terms were assessed. Data was analyzed using STATA 12.0 (StataCorp LP, College Station, TX) and SAS 9.4 (SAS Institute, Cary, NC).

## Results

### Assay validation

The calibration curves showed excellent linear regression parameters for over two months period; 25-OH-D2 showed r>0.9999, slope and intercept of 0.0306 and 0.0046 respectively, and 25-OH-D3 showed r>0.9999, slope and intercept of 0.0327 and 0.0113 respectively. The analytical measurement ranges were 3.5–114.6 ng/mL and 4.1–121.8 ng/mL for 25-OH-D2 and 25-OH-D3 respectively, which compared well to the manufacturer findings (11). The limit of quantitation for either 25-OH-D2 and 25-OH-D3 was 3.4 ng/mL. Recovery studies using pooled serum samples as matrix showed a recovery ranging from 90–110%. Table 1 shows the intra-precision of Cascadion analyzer for 92 samples. The %CV, %bias, and SE ranged 1.16% to 3.80%, -1.60% to 0.90%, and 0.04 to 0.23 respectively for the three control levels, which were within the acceptable criteria of ±8.0% and ±15% for %CV and %bias respectively. No interference was observed from hemolysis, lipemia and bilirubin. All samples spiked with interfering compounds showed a bias of <10%. The Vitamin D assay passed proficiency evaluations from two different CAP kits. Three samples of the CAP Total Vitamin D kit had bias results of -9.88%, -12.86%, and -10.15% respectively for tD. Three samples of CAP Accuracy-based Vitamin D samples had bias of -3.10%, -4.00%, and -8.39% respectively for tD; and bias of -6.09%, -4.00% and 0.75% respectively for 25-OH-D3.

**Table 1:** Precision results of 25-OH-D2, 25-OH-D3, and tD control levels.

Control	Compound	Expected (ng/mL)	Mean (ng/mL)	SD^a^	%CV^b^	%Bias	SE^c^
**1**	25-OH-D2	9.20	9.30	0.35	3.80	0.90	0.04
	25-OH-D3	10.10	10.08	0.36	3.52	0.67	0.04
	tD	19.30	19.45	0.36	1.83	0.78	0.04
**2**	25-OH-D2	28.00	28.04	0.94	3.36	0.14	0.10
	25-OH-D3	30.30	29.55	0.72	2.44	-0.92	0.08
	tD	58.30	57.59	0.83	1.44	-1.22	0.09
**3**	25-OH-D2	83.80	82.79	2.25	2.72	-1.20	0.23
	25-OH-D3	89.30	87.87	1.70	1.94	-1.60	0.18
	tD	173.10	170.66	1.98	1.16	-1.41	0.21

Precision result of 92 Cascadion 25-hydroxyvitamin D control levels. Data were acquired over the period of 8/3/2020 to 12/4/2020. Expected and mean values are in ng/mL.

^a^Standard deviation

^b^Coefficient variance percentage.

^c^Standard error.

**Fig 1:**
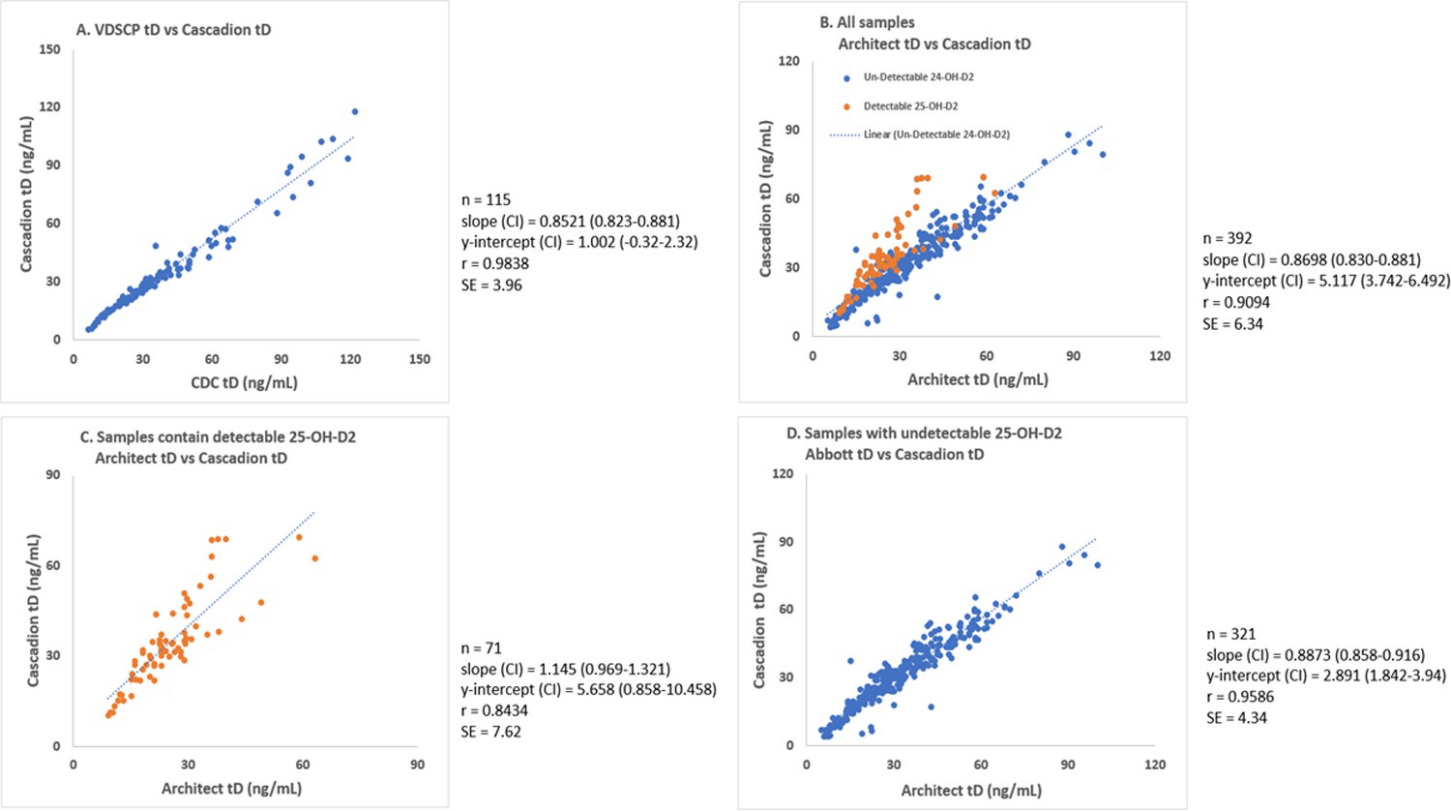
Linear plots and regression parameters. Correlation of Cascadion tD results to A) VDSCP tD B) Architect tD of all patient samples C) Architect tD of samples contain detectable 25-OH-D2 and D) Architect tD of samples with undetectable 25-OH-D2.

The correlation of tD levels using VDSCP samples with Cascadion is shown in Fig 1A–1D. The tD had a best linear fit r = 0.983 for y = 0.852x + 1.002 with a standard error of 3.96 ng/ml for 115 VDSCP samples from the CDC. The patient samples correlation is shown in Fig 1B–1D. As shown in Fig 1B, the patient samples did not correlate as well as VDSCP samples. A closer look at the data revealed that Architect immunoassay under-reported tD levels in patients with detectable 25-OH-D2, resulting in a regression co-efficient of 0.909 and a standard error and average bias of 6.34 ng/ml and -6.47% respectively for a concentration range 5–100 ng/mL. Fig 1C shows the correlation between patient samples containing detectable levels of 25-OH-D2. Patient samples with undetectable levels of 25-OH-D2 correlated well with LCMS results as shown in Fig 1D. These data clearly show that immunoassay underestimated tD levels in patients with Vitamin D2 as a measurable component of total Vitamin D. Results by immunoassay had an average bias of -38.44% as compared to results by LCMS.

Levels of 25-OH-D2 were undetectable in 88.5% of patients, which was inadequate for statistical analysis. 25-OH-D3 and tD in 122 samples from patients without history of COVID-19 infection showed positively skewed Gaussian distribution. When comparing the AUCs for 25-OH-D3 and tD for the mortality, 25-OH-D3 had AUC = 0.660 vs tD AUC = 0.608; however, Delong’s test did not conclude that it was significantly higher (p = 0.291). When comparing sensitivity and specificity, the insufficiency cutoff for 25-OH-D3 has a sensitivity of 52.4% and specificity of 70.2%. The tD insufficiency cutoff had sensitivity of 61.9% and specificity of 49.3%, and for deficiency, sensitivity and specificity were 61.9% and 49.3%, respectively. The insufficiency cutoff for immunoassay had sensitivity of 81.0% and specificity of 31.3%.

Table 2 shows the demographic characteristics of the COVID-19 and non-COVID-19 groups. COVID-19 positive patients were 9.7 years older than non-COVID-19 patients on average (p<0.001). There were more patients in the 81–100 years of age range in COVID-19 positive category (p = 0.008). The ten most common coexisting risk factors are listed in order from high to low prevalence in our study population. Among these, 5 conditions that differed between the two groups were tobacco use, respiratory failure or distress, pneumonia, sepsis, and encephalopathy. Obesity was more prevalent in the COVID-19 patients than the control group (p = 0.001). The mean tD level was significantly higher in the COVID-19 negative than positive group (0.045). The COVID-19 positive group had a higher number of patients with tD less than 20 ng/mL (0.001). The 25-OH-D3 level did not differ significantly (p = 0.195).

**Table 2:** The demographics of study patients.

Demographics	All Sample	COVID-19 positive	COVID-19 negative	p
Number of patients (%)	210	88	122	
Female (%	139 (66.2)	53 (60.2)	86 (70.5)	0.120
Mean age, year (CI^b^)	56.6 (54.0–59.3)	62.3 (58.3–66.2)	52.6 (49.2–55.9)	<0.001*
Age 18–20 years (%)	8 (3.8)	2 (2.3)	6 (4.9)	0.333
Age 21–40 years (%)	37 (17.6)	11 (12.5)	26 (21.3)	0.099
Age 41–60 years (%)	65 (31.0)	23 (26.1)	42 (34.4)	0.200
Age 61–80 years (%)	80 (38.1)	38 (43.2)	42 (34.4)	0.196
Age 81–100 years (%)	20 (9.5)	14 (15.9)	6 (4.9)	**0.008**
Coexisting risk factors (%)				
Hypertension	103 (49.0)	45 (51.1)	58 (47.5)	0.608
Tobacco use	92 (43.8)	49 (55.7)	43 (35.2)	**0.003**
Hyperlipidemia	57 (27.1)	22 (25.0)	35 (28.7)	0.553
Respiratory failure or distress	47 (22.4)	42 (47.7)	5 (4.1)	<**0.001**
Pneumonia	42 (20.0)	38 (43.2)	4 (3.3)	<**0.001**
Diabetes type 2	40 (19.0)	21 (23.9)	19 (15.6)	0.132
Sepsis	28 (13.3)	26 (29.6)	2 (1.6)	<**0.001**
Congestive heart failure	16 (7.6)	10 (11.4)	6 (4.9)	0.081
Atrial fibrillation	16 (7.6)	10 (11.4)	6 (4.9)	0.081
Encephalopathy	14 (6.7)	13 (14.8)	1 (0.8)	<**0.001**
Mean 25-OH-D3, ng/mL (CI)	28.7 (26.6–30.7)	27.0 (23.8–30.3)	29.8 (27.1–32.5)	0.195*
Mean tD, ng/mL (CI)	30.9 (28.8–32.9)	28.4 (25.3–31.6)	32.6 (30.0–35.3)	0.045*
Samples with < 20.0 ng/mL (%)	42 (20.0)	27 (30.7)	15 (12.3)	**0.001**
Samples with < 30.0 ng/mL (%)	102 (48.6)	47 (53.4)	55 (45.1)	0.236
Patients with supplement (%)	119 (56.7)	54 (61.4)	65 (53.3)	0.244
Mean BMI^a^, kg/m^2^ (CI)	30.3 (29.1–31.6)	31.4 (29.2–33.5)	29.6 (28.0–31.1)	0.185
BMI ≥ 30 kg/m^2^ (%)	111 (52.9)	58 (65.9)	53 (43.4)	**0.001**

Characteristics of COVID-19 positive and negative patients in the study. Mean p-values* were determined by t-test, and categorical p-values were determined by x^2^-test of proportions. Bolded p-value numbers are statistically significant p<0.050.

^a^Body mass index (kg/m^2^).

^b^95% confidence interval.

**Fig 2:**
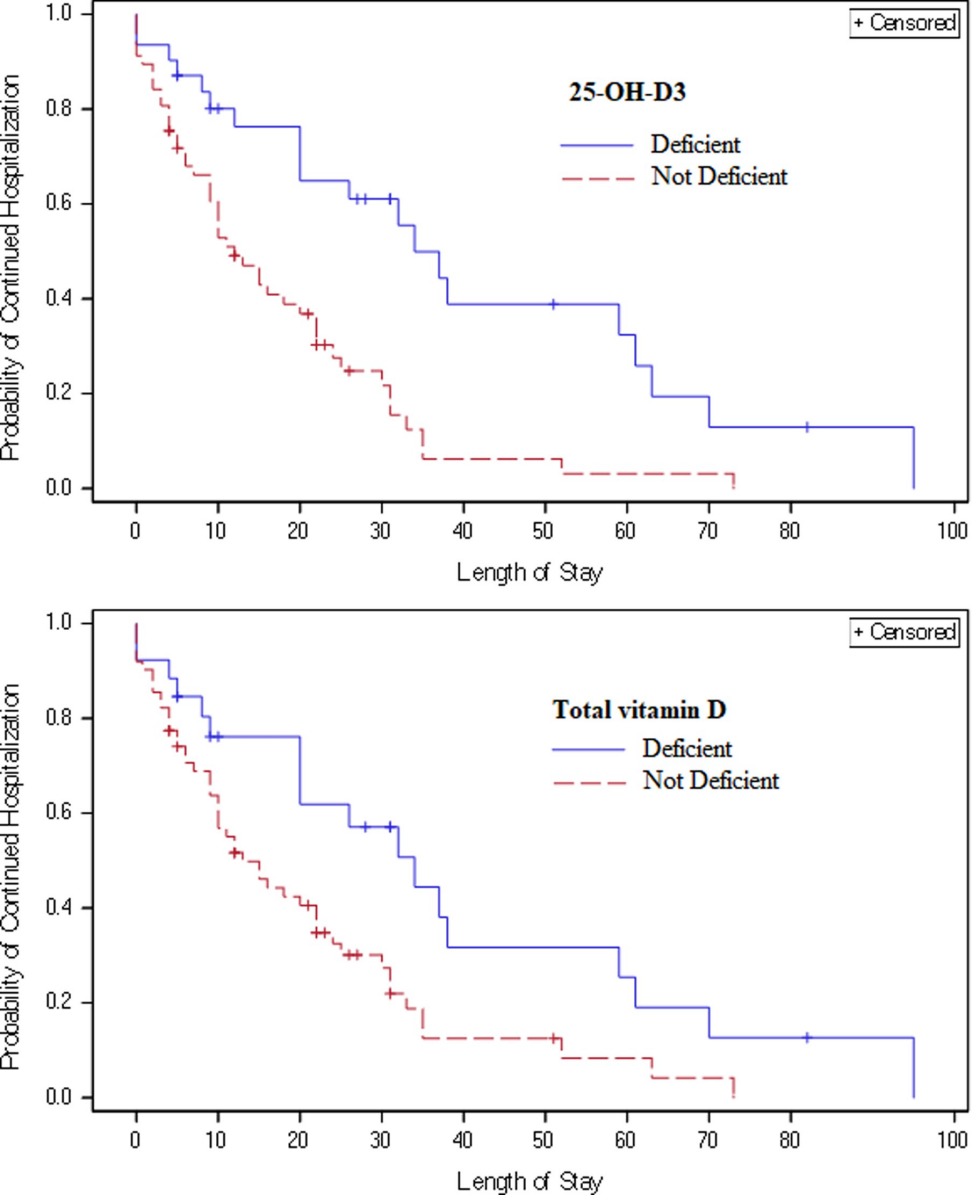
Kaplan-Meier curves for hospital discharge. Unadjusted Kaplan-Meier curves for hospital discharge by 25-OH-D3 and tD levels above and below cutoffs. For 25-OH-D3, there were 11 patients censored at the time of death with deficient levels and 10 with non-deficient levels. For tD, 8 and 13 patients were censored at time of death for deficient and non-deficient levels, respectively.

Table 3 focuses on COVID-19 positive patients deficient of 25-OH-D3 by LCMS, and deficient and insufficient of tD by LCMS and immunoassay. The deficient and insufficient cutoffs were used according to universal consensus accepted by US Endocrine Society for normal bone health. There was a significant association between increased LOS and having deficient and insufficient levels of 25-OH-D3 or tD in all analyses. Time-to-event analysis using Cox proportional hazards models and unadjusted Kaplan-Meier curves (Fig 2) further indicate that COVID-19 positive patients who had deficient levels of 25-OH-D3 (HR for discharge = 0.26 (CI: 0.14–0.49), p<0.001) or tD (HR = 0.37 (CI: 0.20–0.59), p = 0.010) had longer risk-adjusted LOS than those with non-deficient (≥20 ng/mL) levels.

**Table 3:** Analyses of the cutoff levels versus ventilation, mortality, and hospital LOS.

**25-OH-D3**	**A1. LCMS** ^d^			**A2. LCMS**					
	**<20 ng/mL**	**≥20 ng/mL**	**p**	**<30 ng/mL**	**≥30 ng/mL**	**p**			
**Ventilation** (%)									
Unadjusted	21 (67.7)	29 (50.9)	0.127	34 (66.7)	16 (43.2)	**0.029**			
Risk-adjusted OR^a^	2.12	reference	0.127	2.72	reference	**0.034**			
(CI)	(0.81–5.59)			(1.08–6.85)					
**Mortality** (%)									
Unadjusted	11 (35.5)	10 (17.5)	0.059	15 (29.4)	6 (16.2)	0.152			
Risk-adjusted OR	5.29	*reference*	**0.008**	3.83	reference	**0.028**			
(CI)	(1.53–18.24)	* *		(1.16–12.7)					
**Average LOS** ^b^									
Unadjusted	29.8	14.5	**0.003**	25.0	12.9	**0.006**			
(CI)	(20.6–38.9)	(10.8–18.2)		(18.6–31.2)	(8.7–17.1)				
Risk-adjusted HR^c^	0.26	*reference*	**<0.001**	0.35	reference	**<0.001**			
(CI)	(0.14–0.49)			(0.20–0.60)					
**Total 25-OH Vitamin D**	**B1. LCMS**			**B2. LCMS**			**B3. Immunoassay**		
	**<20 ng/mL**	**≥20 ng/mL**	**p**	**<30 ng/mL**	**≥30 ng/mL**	**p**	**<30 ng/mL**	**≥30 ng/mL**	**p**
**Ventilation** (%)									
Unadjusted	18 (69.2)	32 (51.6)	0.128	32 (68.1)	18 (43.9)	**0.022**	38 (60.3)	12 (48.0)	0.293
Risk-adjusted OR	2.23	*reference*	0.125	2.75	*reference*	**0.031**	1.54	*reference*	0.397
(CI)	(0.80–6.24)			(1.10–6.90)			(0.57–4.14)		
**Mortality** (%)									
Unadjusted	8 (30.8)	13 (21.0)	0.325	13 (27.7)	8 (19.5)	0.371	17 (27.0)	4 (16)	0.276
Risk-adjusted OR	3.12	*reference*	0.069	2.7	*reference*	0.087	3.68	*reference*	0.055
(CI)	(0.92–10.64)	* *		(0.87–0.84)			0.87–13.90	* *	
**Average LOS**									
Unadjusted	28.4	16.3	**0.045**	23.7	15.5	**0.047**	23.8	10.1	**0.001**
(CI)	(17.9–38.9)	(12.4–20.2)		(17.2–30.3)	(10.5–20.4)		(18.4–29.1)	(5.7–14.4)	
Risk-adjusted HR	0.37	*reference*	**0.002**	0.5	*reference*	**0.010**	0.27	*reference*	**<0.001**
(CI)	(0.20–0.69)			(0.29–0.85)			(0.14–0.51)		

Evaluation of mortality, need for ventilation, and LOS for COVID-19 positive patients at universal cutoff for deficiency at 20 ng/mL (A1 & B1) and insufficiency at 30 ng/mL (A2, B2 & B3). LOS differed statistically significant by all analyses. Odds of mortality only differed significantly when using 25-OH-D3 deficiency and insufficiency cutoffs after risks adjusted for LCMS results. Need for ventilation only differed significantly when using 25-OH-D3 and tD insufficiency cutoff for LCMS results. Unadjusted p-values were determined by Wilcoxon rank sum test for LOS and x^2^-test for mortality. Adjusted analysis controlled for age, sex, and BMI. Logistic regression was used for mortality, and Cox proportional hazards were used for LOS.

^a^Odds ratio.

^b^Hospital Length of Stay (days).

^c^Hazard ratio.

^d^Liquid chromatography tandem mass spectrometry.

Ventilation requirement and mortality between the insufficient (<30 ng/mL) and sufficient (≥30 ng/mL) groups were not significantly different using immunoassay results. However, considering the LCMS results, the mortality odds ratio was significantly different between the 25-OH-D3 deficient (<20 ng/mL) and non-deficient (≥20 ng/mL) as well as the insufficient (<30 ng/mL) and sufficient (≥30 ng/mL) groups (Table 3A1 and 3A2) after risk-adjustment for age, sex and BMI. The need for ventilation during hospitalization also differed significantly using insufficient and not deficient cutoffs for both 25-OH-D3 and tD by LCMS (Table 3A2 & 3B2).

## Discussion

This is the first and probably only study in North America using a random access LCMS analyzer to measure 25-OH-D2 and 25-OH-D3 in COVID-19 positive patients and correlate the results to disease severity. Our data indicate that 25-OH-D3 or tD results could predict the LOS, ventilation need, and mortality odds of hospitalized COVID-19 patients. Hospitalized COVID-19 patients with deficient or insufficient levels of either 25-OH-D3 or tD stayed an average of 12.9 days longer in the hospital for disease monitoring and treatment. At the 25-OH-D3 cutoff at 20 ng/mL for deficiency, the risk-adjusted mortality odds were more than 5-fold observed in the deficient group (p = 0.008). At the tD cutoff at 30 ng/mL for insufficiency, vitamin D insufficient patients required more frequently ventilator support, 2.75-fold more than those patients having tD levels ≥30 ng/mL (p = 0.031) after risk-adjustment for patient characteristics.

Quantitative results from LCMS analysis showed high agreement to the VDSCP results (Fig 1A). All CAP proficiency results were within the acceptable standard deviation index (SDI) limits. The LCMS has higher analytical specificity for the two components of tD when compared to immunoassay method, which has been reported to underestimate 25-OH-D2, and was again shown to do so in our results. This bias is recognized by the immunoassay manufacturers and LCMS confirmation is recommended whenever tD levels do not correlate with clinical symptoms or in samples with high triglyceride [16]. It also explains why the measured levels of tD by immunoassay did not correlate well with LCMS, as is seen from our patient correlation data. The regression coefficient, slope and intercept improved significantly when data from patients with detectable levels of 25-OH-D2 were removed (Fig 1D).

We are unsure as to why lower levels of 25-OH-D3 and tD are linked with longer LOS and, in risk-adjusted odds ratio, higher in-hospital ventilation requirements and mortality, and by extension increased severity in hospitalized COVID-19 patients. Our finding is, however, similar to previous reports [6, 17–19]. The degree of low levels of vitamin D has been shown to be inversely correlated with severity of COVID-19 infection (odds ratio 1.95, p = 0.029), hospitalization rates (odds ratio 1.77, p = 0.026) and increased LOS (regression coefficient = 0.47, p = 0.0001) [21]. Numerous mechanisms linking COVID-19 risk and severity to vitamin D deficiency have been hazarded [17, 18]. Vitamin D is known to reduce cytokine production by increasing the innate immune system and lowering the adaptive immune response to viral load. Vitamin D inhibits the differentiation and maturation of MHC Class II, CD40, CD80 and CD86. The activation of T cells by the dendritic cells is also inhibited by low Vitamin D levels [20, 21]. The prevailing school of thought is that low levels of tD are i) associated with a decreased number of the regulatory T lymphocytes, which are reported to be markedly decreased in severe cases of COVID-19 [22, 23] and ii) Vitamin D deficiency could aggravate the reported thrombotic complications in COVID-19 patients, since normal levels of Vitamin D are essential for regulating thrombotic pathways. Increased production of IL-10 is seen with Vitamin D treatment increasing the regulatory T cells [24]. At the very least, it can be concluded that Vitamin D is a crucial nutrient and its involvement in recovery of COVID-19 patients is multifaceted and should be investigated through well-designed clinical trials.

## Conclusion

In this study we have shown that i) 25-OH-D3 or tD levels are inversely correlated to hospital LOS, ventilation requirements, and odds of in-hospital mortality and ii) differentiating 25-OH-D2 and 25-OH-D3 by LC-MS/MS could identify patients who may need Vitamin D supplements to achieve/maintain healthy levels. Further research, including randomized controlled trials of Vitamin D supplementation, are needed to establish the nature of the associations between Vitamin D levels and risk for COVID-19, and risk of adverse outcomes in patients diagnosed with COVID-19. Considering the rapid spread of COVID-19 worldwide, even a moderately beneficial effect of Vitamin D3 in reducing the risk of infection and disease progression could yield improved outcomes for COVID-19 positive patients as Vitamin D3 is readily available, easily administered, and have little risk of adverse effects.

### Limitations

This was an observational study, and so we cannot rule out the possibility that there may be unmeasured confounders impacting the associations observed between Vitamin D levels and COVID-19 infection and outcomes. While we adjusted for the patient demographic and clinical history factors, other factors such as socioeconomic depravation or ethnicity, which may affect LOS, mortality, and/or Vitamin D levels, could not be account for. We also cannot determine whether the lower Vitamin D levels observed among hospitalized patients testing positive for COVID-19 compared to outpatients testing negative, or among hospitalized COVID-19 patients with poorer outcomes (longer stay, in-hospital mortality) were secondary to these patients being in the acute phase of disease rather than a causative effect. Low case numbers and patient demographics may not reflect the true situation for different patient characteristics and locale; a large-scale study is indicated to confirm these results. Multiple stratified thresholds were not defined and may be necessary in evaluating various stages of vitamin D deficiency and defining risks for increased severity. Additionally, while we used traditional statistical analyses found in many similar studies, these methods can be limited by their assumptions and violations of those assumptions that commonly occur with real-world data.

## Supporting information

S1 Table Full models for adjusted evaluation of mortality, need for ventilation, and LOS. Full models for adjusted evaluation of mortality, need for ventilation, and LOS for COVID-19 positive patients at deficiency and insufficiency cutoffs for 25-OH-D3 and tD. Logistic regression was used for mortality and ventilation, and Cox proportional hazards was used for LOS.
(DOCX)

S1 Data Study data. All patient results and demographics information.
(XLSX)
