# Supplementary material for: 25-hydroxyvitamin D is a predictor of COVID-19 severity of hospitalized patients
Source: PLoS One. 2022 May 3;17(5):e0268038. doi: 10.1371/journal.pone.0268038 (PMC9064100; doi:10.1371/journal.pone.0268038)
Supplement: S1 Table — Full models for adjusted evaluation of mortality, need for ventilation, and LOS for COVID-19 positive patients at deficiency and insufficiency cutoffs for 25-OH-D3 and tD. Logistic regression was used for mortality and ventilation, and Cox proportional hazards was used for LOS. (DOCX) [file pone.0268038.s001.docx]

**S3 Table**. **Full models for adjusted evaluation of mortality, need for ventilation, and LOS.**

|  | **Ventilation** | | | **Mortality** | | | **LOS**^d^ **(days)** | | |
| --- | --- | --- | --- | --- | --- | --- | --- | --- | --- |
|  | Odds Ratio | 95% CI^c^ | p | Odds Ratio | 95% CI | p | Hazard Ratio | 95% CI | p |
| **25-OH-D3 by LCMS**^a^ **(CO**^b^ **= 20 ng/mL)** | 2.12 | (0.81 - 5.59) | 0.127 | 5.29 | (1.53 - 18.24) | 0.008 | 0.26 | (0.14 - 0.49) | <0.001 |
| Age | 1.01 | (0.98 - 1.04) | 0.473 | 1.07 | (1.02 - 1.12) | 0.003 | 0.97 | (0.96 - 0.99) | 0.002 |
| Sex F vs M | 0.51 | (0.2 - 1.29) | 0.157 | 1.13 | (0.37 - 3.46) | 0.836 | 0.86 | (0.5 - 1.46) | 0.566 |
| BMI | 1.04 | (0.99 - 1.1) | 0.136 | 0.99 | (0.93 - 1.06) | 0.778 | 0.99 | (0.96 - 1.01) | 0.318 |
| **25-OH-D3 by LCMS (CO = 30 ng/mL)** | 2.72 | (1.08 - 6.85) | 0.034 | 3.83 | (1.16 - 12.7) | 0.028 | 0.35 | (0.2 - 0.6) | <0.001 |
| Age | 1.01 | (0.99 - 1.04) | 0.384 | 1.07 | (1.02 - 1.12) | 0.003 | 0.98 | (0.96 - 0.99) | 0.008 |
| Sex F vs M | 0.53 | (0.21 - 1.36) | 0.189 | 1.15 | (0.38 - 3.45) | 0.801 | 0.94 | (0.55 - 1.58) | 0.802 |
| BMI | 1.04 | (0.99 - 1.1) | 0.153 | 0.99 | (0.94 - 1.06) | 0.847 | 0.99 | (0.96 - 1.01) | 0.316 |
| **Total 25-OH Vitamin D LCMS (CO = 20 ng/mL)** | 2.23 | (0.8 - 6.24) | 0.125 | 3.12 | (0.92 - 10.64) | 0.069 | 0.37 | (0.20 - 0.69) | 0.002 |
| Age | 1.01 | (0.98 - 1.04) | 0.456 | 1.07 | (1.02 - 1.11) | 0.004 | 0.98 | (0.96 - 0.99) | 0.006 |
| Sex F vs M | 0.52 | (0.21 - 1.3) | 0.161 | 1.09 | (0.37 - 3.22) | 0.875 | 0.87 | (0.51 - 1.48) | 0.606 |
| BMI | 1.04 | (0.99 - 1.1) | 0.136 | 0.99 | (0.93 - 1.06) | 0.855 | 0.99 | (0.96 - 1.01) | 0.275 |
| **tD by LCMS**  **(CO = 30 ng/mL)** | 2.75 | (1.10 - 6.90) | 0.031 | 2.70 | (0.87 - 8.44) | 0.087 | 0.50 | (0.29 - 0.85) | 0.010 |
| Age | 1.01 | (0.99 - 1.04) | 0.379 | 1.07 | (1.02 - 1.11) | 0.004 | 0.98 | (0.97 – 1.00) | 0.022 |
| Sex F vs M | 0.55 | (0.22 - 1.41) | 0.214 | 1.16 | (0.39 - 3.40) | 0.794 | 0.91 | (0.54 - 1.54) | 0.724 |
| BMI | 1.04 | (0.98 - 1.10) | 0.166 | 0.99 | (0.94 - 1.06) | 0.838 | 0.99 | (0.96 - 1.01) | 0.303 |
| **tD by Immunoassay (CO = 30 ng/mL)** | 1.54 | (0.57 - 4.14) | 0.397 | 3.68 | (0.97 - 13.9) | 0.055 | 0.27 | (0.14 - 0.51) | <0.001 |
| Age | 1.01 | (0.98 - 1.04) | 0.517 | 1.07 | (1.02 - 1.11) | 0.003 | 0.98 | (0.96 - 0.99) | 0.006 |
| Sex F vs M | 0.55 | (0.22 - 1.38) | 0.205 | 1.21 | (0.41 - 3.62) | 0.729 | 1.04 | (0.61 - 1.78) | 0.887 |
| BMI | 1.04 | (0.99 - 1.1) | 0.130 | 0.99 | (0.93 - 1.05) | 0.758 | 0.99 | (0.96 - 1.01) | 0.271 |

Full models for adjusted evaluation of mortality, need for ventilation, and LOS for COVID-19 positive patients at deficiency and insufficiency cutoffs for 25-OH-D3 and tD. Logistic regression was used for mortality and ventilation, and Cox proportional hazards was used for LOS.

^a^LCMS = liquid chromatography tandem mass spectrometry.

^b^Cutoff concentration level.

^c^95% Confidence Interval.

^d^Hospital Length of Stay (days).
